# Treatment of negative dysphotopsia with supplementary implantation of a sulcus-fixated intraocular lens

**DOI:** 10.1007/s00417-015-3029-8

**Published:** 2015-05-07

**Authors:** Natalia Y. Makhotkina, Tos T. J. M. Berendschot, Henny J. M. Beckers, Rudy M. M. A. Nuijts

**Affiliations:** University Eye Clinic, Maastricht University Medical Centre, P. Debyelaan 25, 6202 AZ Maastricht, The Netherlands

**Keywords:** Negative dysphotopsia, Pseudophakia/complications, Sulcus-fixated intraocular lens

## Abstract

**Purpose:**

Our aim was to evaluate the resolution of negative dysphotopsia after supplementary implantation of a sulcus-fixated intraocular lens (IOL).

**Methods:**

This was a retrospective case series. Patients with severe negative dysphotopsia were treated with supplementary implantation of the Rayner Sulcoflex Aspheric (653 L) IOL. Primary outcome measurements were subjectively reported complaints of dysphotopsia, best corrected distance visual acuity (CDVA), iris-IOL distance, anterior chamber depth (ACD) and volume (ACV), angle opening distance and trabecular-iris space area at 500 and 750 μm.

**Results:**

A Rayner Sulcoflex IOL was implanted in seven patients (nine eyes) with negative dysphotopsias. Symptoms resolved completely in six eyes, partially in one eye and remained unchanged in two eyes. We did not find any significant changes in CDVA. Angle opening distance, ACD, ACV and iris-IOL distance reduced significantly after Sulcoflex IOL implantation.

**Conclusions:**

Supplementary implantation of a Sulcoflex IOL can successfully treat negative dysphotopsia. The decrease in anterior segment dimensions in combination with the displacement of light rays by the rounded edges of a Sulcoflex IOL may contribute to the resolution of symptoms.

## Introduction

Unwanted optical phenomena such as negative and positive dysphotopsias are well known side effects after cataract surgery [1]. Negative dysphotopsia is defined as the perception of a shadow obscuring the temporal field of vision, while positive dysphotopsia is characterised by halos, arcs or streaks around point light sources [2, 3].

In the majority of cases, dysphotopsias resolve or diminish over time. Therefore “watchful waiting” and reassurance are reasonable initial treatment strategies. However, in 0.2 to 1 % of pseudophakic patients severe symptoms will persist [2, 4] and additional surgery may be required.

Implantation of a secondary intraocular lens (IOL) has been proposed as an option to alleviate negative dysphotopsias [5, 6]. Partial or complete resolution of symptoms has been reported after supplementary implantation of a Sulcoflex 653 L IOL (Rayner Intraocular Lenses Ltd, East Sussex, UK) in one eye [6], AQ5010V IOLs (STAAR Surgical Company, Monrovia, CA) in six eyes [5] and a Clariflex IOL (Abbott Medical Optics Inc., Santa Ana, CA) in one eye [5]. In this study, we report a case series of patients with severe negative dysphotopsias who were treated with supplementary implantation of a Sulcoflex IOL.

## Materials and methods

Files of patients with negative dysphotopsias who underwent supplementary implantation of a Sulcoflex IOL were reviewed retrospectively. In our hospital all clinical data may be used for research, unless a patient has given a written objection. Further, local medical ethical committee requires no approval for retrospective studies.

**Fig. 1:**
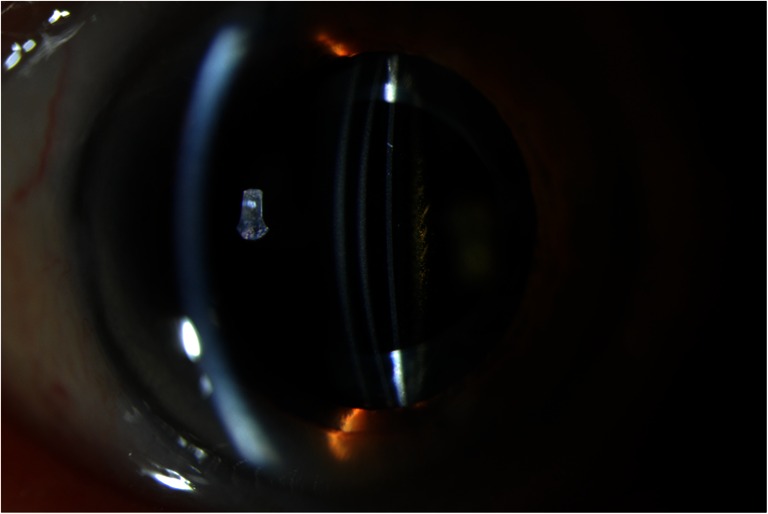
A slit-lamp photograph of a supplementary IOL: a cross-sectional view (Case 1)

All patients underwent uneventful phacoemulsification with IOL implantation in the capsular bag. Secondary implantations were performed by two experienced surgeons (R.N. and H.B.) at the University Eye Clinic in Maastricht. A peripheral iridotomy was made to prevent postoperative intraocular pressure (IOP) spikes. A Sulcoflex Aspheric (653 L) IOL with powers varying from −0.5 to 0.5D was used in all cases and placed in the ciliary sulcus (Fig. 1). Complaints of dysphotopsia were noted before surgery and at each follow-up visit. Evaluation included uncorrected (UDVA) and corrected (CDVA) distance visual acuity, manifest refraction, Goldman applanation tonometry, slit-lamp examination, Scheimpflug photography (Pentacam, Oculus Optikgeräte GmbH, Wetzlar, Germany) and anterior segment optical coherence tomography (Visante, Carl Zeiss Meditec, Inc, Dublin, CA and Casia, SS-1000, Tomey Corporation, Nagoya, Japan).

## Results

A Sulcoflex IOL was implanted in seven patients (nine eyes) with negative dysphotopsias (Table 1). Two patients also had positive dysphotopsias. The mean age of the patients was 63.0 ± 6.6 years. Negative dysphotopsias resolved completely in six eyes, partially in one eye and remained unchanged in two eyes.

**Table 1:** Preoperative characteristics and symptoms following Sulcoflex IOL implantation

Case	Sex	Age, years	Eye	IOL	Dysphotopsia	Follow-up, months	Symptom course			Fellow eye dysphotopsia^a^
							1 week	1 month	Last visit	
1	M	69	R	AcrySof SN60WF	Negative	8	The black crescent ↓	The black crescent ↓↓↓	The black crescent completely resolved	
			L	Acrysof SN60WF	Negative	5	The black crescent completely resolved			
2	F	67	L	AcrySof SN60WF	Negative	8	The black bar is gone	The black bar recurred; however, it was smaller and was not bothersome anymore		No
3	M	52	L	Tecnis ZCB00	Negative	5	The black bar completely resolved			NR
4	F	61	L	AcrySof SN60WF	Negative	22	The black bar completely resolved			Yes, negative
5	F	69	R	Tecnis ZCB00	Combined	8	The dark crescent completely resolved			
			L	Tecnis ZCB00	Negative	3	The dark crescent completely resolved			
6	F	53	L	Sensar AR40e	Combined	5	Black bar persisted, halos and glare increased		The Sulcoflex IOL was explanted. The symptoms returned to the preoperative level.	Yes, positive
7	F	70	R	Acrysof SN60WF	Negative	4	The black bar ↓	The black bar recurred and was still bothersome		No

*IOL* intraocular lens; *F* female; *M* male; *L* left eye; *R* right eye; *NR* not relevant since the right eye did not undergo phacoemulsification; ↓, decreased

^a^IOLs implanted in fellow eyes were similar to IOL type in affected eyes

There was no significant change in CDVA, spherical equivalent refraction (SE) and IOP after surgery (Table 2). Anterior chamber depth (ACD) and volume were significantly reduced. All iridocorneal angle measurements were reduced, and this was significant for angle opening distances at 500 and 750 μm. Implantation of the supplementary IOLs caused a small posterior movement of the primary IOLs, as the distance between the posterior corneal surface and the primary IOL increased significantly by 2 % (range 0 to 3 %).

**Table 2:** Preoperative and postoperative patient characteristics

Mean ± SD	Preoperative	Postoperative	*p*-value (paired samples *T*-test)
CDVA, log MAR^a^	−0.02 ± 0.04	−0.05 ± 0.08	0.39
SE, D	0.13 ± 0.23	−0.00 ± 0.36	0.43
IOP, mm Hg	12.3 ± 1.8	13.3 ± 2.5	0.24
AS-OCT (7 eyes)			
AOD 500, mm			
Nasal	0.60 ± 0.20	0.50 ± 0.17	< 0.05
Temporal	0.64 ± 0.21	0.51 ± 0.17	< 0.05
AOD 750, mm			
Nasal	0.91 ± 0.23	0.77 ± 0.18	< 0.01
Temporal	0.97 ± 0.32	0.80 ± 0.25	< 0.05
TISA 500, mm			
Nasal	0.22 ± 0.06	0.19 ± 0.07	0.07
Temporal	0.22 ± 0.07	0.18 ± 0.07	0.24
TISA 750, mm			
Nasal	0.41 ± 0.12	0.35 ± 0.11	0.07
Temporal	0.42 ± 0.14	0.34 ± 0.12	0.05
Iris-IOL distance, mm	0.61 ± 0.11	0.01 ± 0.02	< 0.01
ACD, mm	4.16 ± 0.36	3.20 ± 0.28	< 0.01
Cornea-IOL distance, mm^b^	0.56 ± 0.11	0.47 ± 0.02	< 0.05
Scheimpflug photography (8 eyes)			
ACD, mm	4.37 ± 0.48	3.13 ± 0.23	< 0.01
ACV, mm^3^	176 ± 28	154 ± 25	< 0.01

*ACD* anterior chamber depth; *ACV* anterior chamber volume; *AOD* angle opening distance; *CDVA* best corrected distance visual acuity; *AS-OCT* anterior segment optical coherence tomography; *D* diopter; *IOL* intraocular lens; *SE* spherical equivalent (Sphere+1/2 Cylinder); *SD* standard deviation; *TISA* trabecular iris space area

^a^One eye with late AMD and CDVA of 20/2000 before Sulcoflex implantation and CDVA 20/400 after surgery was excluded from analysis

^b^Distance between the posterior corneal surface and the primary IOL

Explantation of the Sulcoflex IOL was performed in one patient (case 6) who reported increased positive and unchanged negative dysphotopsia after surgery. After explantation of the Sulcoflex IOL, symptoms returned to the preoperative level. In one patient (case 4), a small anterior chamber hemorrhage occurred after peripheral iridotomy. No other complications, e.g., iris chafing, inflammation or IOP elevation occurred during follow-up.

## Discussion

The current treatment options for severe persistent negative dysphotopsia are IOL exchange with placement of a secondary IOL in the bag or in the ciliary sulcus, implantation of a supplementary IOL, reverse optic capture and Nd: YAG anterior capsulectomy; however, in some cases the symptoms may persist after treatment [2, 4–12]. We have shown that supplementary implantation of a Sulcoflex IOL can successfully treat negative dysphotopsias. Because of the complexity of dysphotopsia pathogenesis, in several cases symptoms may persist or only partially resolve. Negative dysphotopsia occurs with IOLs of different materials [2, 5, 10, 11] with both rounded and squared edges [2, 10, 13]. All our patients had acrylic IOLs with 6.0 mm optics and squared or frosted edges. Davison suggested that some patients might develop an unique interaction between the optical pathways of the eye and the IOL [4]. One possible mechanism is the reflection of light rays between the IOL edges and the anterior capsulorhexis, which can be successfully treated with anterior Nd:YAG capsulectomy, reverse optic capture, or by covering of the anterior capsulorhexis with a sulcus IOL [5, 8, 9].

A large distance between the anterior surface of the IOL and the posterior iris surface may also play a role [3, 14], as the reduction of this distance by implantation of an IOL in the sulcus can eliminate the symptoms [15]. This distance was reduced in all our cases; however, in two cases dysphotopsia did not improve. Persistence of dysphotopsia in cases with a shallow posterior chamber were also reported by Masket et al. [5].

**Fig. 2:**
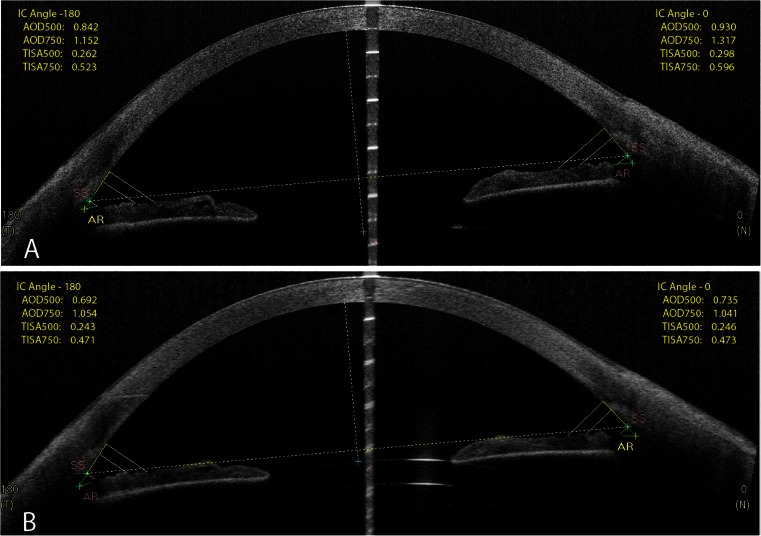
AS-OCT scans with angle analysis before (**a**) and after (**b**) supplementary IOL implantation (Case 1). AOD500, angle opening distance at 500 μm; AOD750, angle opening distance at 750 μm; AR, bottom of the angle, IC, iridocorneal angle; SS, scleral spur; TISA500, trabecular-iris space area at 500 μm; TISA750, trabecular-iris space area at 750 μm

The dimensions of the anterior chamber were reduced after secondary surgery (Fig. 2a and b). Increase in the ACD and iridocorneal angle after phacoemulsification may possibly contribute to the development of negative dysphotopsia, and therefore reduction of these parameters by Sulcoflex IOL implantation may be a good strategy. However, the retrospective character of our study does not allow us to establish any causal relationships.

We have found minor posterior movement of the primary IOL which did not cause any significant change in SE, and none of the patients lost more than two lines of CDVA. A supplementary IOL was removed without any complications in a patient, who was dissatisfied with the outcome.

In conclusion, supplementary implantation of the Sulcoflex IOL is a safe and effective treatment of persistent negative dysphotopsia. A Sulcoflex IOL reduces the dimensions of the anterior and posterior chambers, covers the anterior capsulorhexis, and may refract or reflect light rays by its surfaces and rounded edges. All these mechanisms may reduce the intensity of the photic images on the retina and contribute to the development of neuroadaptation.
